# DUOX1 silencing in lung cancer promotes EMT, cancer stem cell characteristics and invasive properties

**DOI:** 10.1038/oncsis.2016.61

**Published:** 2016-10-03

**Authors:** A C Little, D Sham, M Hristova, K Danyal, D E Heppner, R A Bauer, L M Sipsey, A Habibovic, A van der Vliet

**Affiliations:** 1 Department of Pathology and Laboratory Medicine, University of Vermont, Burlington, VT, USA; 2 Vermont Lung Center, College of Medicine, University of Vermont, Burlington, VT, USA

## Abstract

Dual oxidase 1 (DUOX1) is an oxidant-generating enzyme within the airway epithelium that participates in innate airway host defense and epithelial homeostasis. Recent studies indicate that DUOX1 is suppressed in lung cancers by epigenetic silencing, although the importance of DUOX1 silencing in lung cancer development or progression is unknown. Here we show that loss of DUOX1 expression in a panel of lung cancer cell lines is strongly associated with loss of the epithelial marker E-cadherin. Moreover, RNAi-mediated DUOX1 silencing in lung epithelial cells and the cancer cell line NCI-H292 was found to result in loss of epithelial characteristics/molecular features (altered morphology, reduced barrier function and loss of E-cadherin) and increased mesenchymal features (increased migration, anchorage-independent growth and gain of vimentin/collagen), suggesting a direct contribution of DUOX1 silencing to epithelial-to-mesenchymal transition (EMT), an important feature of metastatic cancer. Conversely, overexpression of DUOX1 in A549 cells was capable of reversing EMT features. DUOX1 silencing in H292 cells also led to enhanced resistance to epidermal growth factor receptor tyrosine kinase inhibitors such as erlotinib, and enhanced levels of cancer stem cell (CSC) markers CD133 and ALDH1. Furthermore, acquired resistance of H292 cells to erlotinib resulted in enhanced EMT and CSC features, as well as loss of DUOX1. Finally, compared with control H292 cells, H292-shDUOX1 cells displayed enhanced invasive features *in vitro* and *in vivo.* Collectively, our findings indicate that DUOX1 silencing in lung epithelial cancer cells promotes features of EMT, and may be strongly associated with invasive and metastatic lung cancer.

## Introduction

The NADPH oxidase dual oxidase 1 (DUOX1) is highly expressed in differentiated airway and alveolar epithelia, and generates H_2_O_2_ in response to various environmental triggers as part of innate protective response against challenges to the airway.^1, 2, 3^ Activation of airway DUOX1 mediates epithelial production of inflammatory mediators and mucus proteins, and promotes cell migration, as critical events in mucosal host defense and maintenance of airway epithelial integrity,^2, 4, 5^ events that largely depend on redox-dependent activation of cell signaling mechanisms involving Src family tyrosine kinases^6, 7^ and epidermal growth factor receptor (EGFR)-dependent pathways.^4, 7, 8^ As increased expression and/or activation of Src and EGFR are well-known features of lung cancer,^9, 10, 11^ it is plausible that lung cancer may also be associated with increased expression or activation of DUOX1. Indeed, production of reactive oxygen species (ROS) from NADPH oxidases (NOX), including DUOX1, has been reported to promote genomic instability and alter signaling pathways involved in carcinogenesis,^12, 13, 14^ and cancer cells commonly produce elevated levels of ROS as potential mediators of mitogenic signaling and metastasis.^15, 16, 17^ Furthermore, expression of certain NOX isoforms is often increased in various cancers, although the distribution and expression levels are highly diverse across cancers, and the biological roles of different NOX isoforms in cancer development and progression are still poorly understood.^12, 14^

In apparent contrast with these concepts, several recent studies demonstrated that DUOX1, as well as its maturation factor, DUOXA1, is frequently silenced in various epithelial cancers, including lung cancer, due to DNA hypermethylation within their promoter regions.^18, 19, 20, 21^ The significance of DUOX1/DUOXA1 silencing in cancer is unclear, and might suggest a role for DUOX1 and/or DUOXA1 as a potential tumor suppressor, potentially by mediating ROS-dependent inhibition of cytokinesis.^22^ DUOX1 suppression may also be relevant for maintenance of cancer stem cells (CSCs), which typically contain reduced ROS levels as an essential feature for maintaining quiescence and self-renewal.^23, 24^ Furthermore, as DUOX1 activation participates in epithelial Src/EGFR-mediated signaling,^6, 7, 8^ DUOX1 silencing might result in altered regulation of these kinases, with potential consequences for development of resistance against tyrosine kinase inhibitors (TKIs). In this regard, responsiveness of non-small-cell lung cancers to EGFR TKI is strongly related to epithelial phenotype^25, 26, 27^ and acquired resistance to EGFR TKI has been associated with epithelial-to-mesenchymal transition (EMT), a critical feature of invasive and metastatic cancers that is associated with poor prognosis in lung cancer.^26, 28, 29, 30^

The present studies were conducted to address the potential association of DUOX1 silencing in lung cancer, with development of EMT and EGFR TKI resistance. Indeed, our results demonstrate that RNAi-mediated DUOX1 silencing in lung epithelial cells and the lung cancer cell line H292 induces loss of epithelial characteristics, increases features of EMT and promotes invasive properties. Conversely, DUOX1 overexpression in lung cancer cells was able to reverse EMT features and enhance epithelial characteristics. DUOX1 silencing was also found to promote EGFR TKI resistance and enhance features of CSCs, suggesting the significance of DUOX1 silencing in lung cancer as a possible indicator of lung cancers with poor prognosis or lack of responsiveness to common anticancer therapy.

## Results

### DUOX1 silencing induces features of EMT

**Figure 1:**
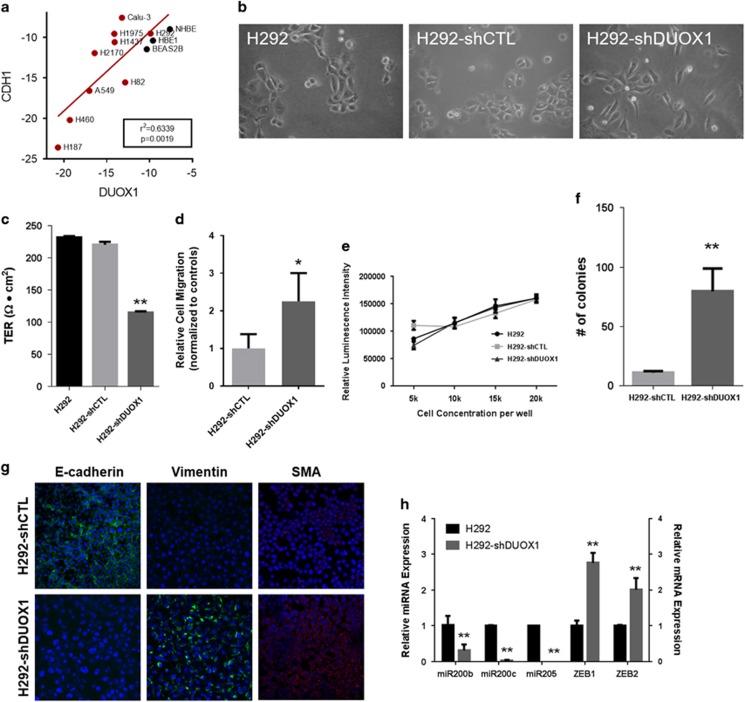
DUOX1 silencing displays functional mesenchymal features. (**a**) Evaluation of DUOX1 and E-cadherin (CDH1) mRNA expression in normal non-cancer airway epithelial cells (black dots) and a panel of lung cancer cell lines (red dots). (**b**) Phase contrast brightfield microscope images taken of host cell line H292, H292 cells transfected with an empty vector (H292-shCTL) and H292 cells transfected with short hairpin RNA targeted against DUOX1 (H292-shDUOX1). (**c**) Trans-epithelial resistance (Ω) characterization of each cell model. (**d**) Cell migration as measured by Donut cell migration assay. (**e**) Cells were seeded at various densities and allowed to grow in culture for 72 h. Post 72 h, cell viability via ATP production was determined in a luminescence assay. (**f**) Cells were grown on soft agar, and colonies counted to assess anchorage-independent growth. (**g**) Fluorescent images of EMT markers; SMA, smooth muscle actin. (**h**) Quantitative real-time PCR results of miR-200 family and transcriptional regulators of E-cadherin, ZEB1/2. *P<0.05 and **P<0.01 were calculated by Student's *t*-test.

We evaluated a panel of non-cancer airway epithelial cells and various lung cancer cell lines for mRNA expression of DUOX1 and DUOX2, and observed a general loss of DUOX1 and DUOX2 in most lung cancer cell lines tested (Figure 1a; Supplementary Figure S1A), with the exception of the pulmonary mucoepidermoid cell line H292 and the lung adenocarcinoma cell line Calu-3, two cancer-derived cell lines that maintain many normal airway epithelial features^8, 31^ and maintain DUOX1 expression. We therefore hypothesized that epithelial DUOX1 expression may associate with epithelial characteristics. Indeed, DUOX1 expression status in a panel of lung cancer cell lines strongly correlated with expression of the epithelial marker E-cadherin (CDH1), suggesting that DUOX1 silencing in lung cancer is associated with loss of epithelial characteristics. To explore this further, we silenced DUOX1 in the DUOX1-expressing cell line H292, by stable transfection with DUOX1-targeted shRNA,^7^ and selected three individual clones for use in subsequent studies. We observed that H292-shDUOX1 cells display altered morphological features compared with normal or control transfected H292 cells (Figure 1b), with loss of cobblestone appearance and intercellular junctions, and a more fibroblast-like cell morphology. Functional analysis also indicated a loss of epithelial characteristics of H292-shDUOX1 cells, with markedly reduced transepithelial electrical resistance (Figure 1c), reflecting loss of epithelial barrier function due to loss of epithelial adherens junctions that are maintained by E-cadherin. H292-shDUOX1 cells also display increased migratory ability, as measured using a recently developed ‘donut' cell migration assay^32^ (Figure 1d; Supplementary Figure S1F), and using a scratch wound assay and a Transwell migration assay^5^ (Supplementary Figures S1C and D). In contrast, DUOX1 deletion did not significantly affect proliferation (Figure 1e; Supplementary Figure S1E). Moreover, H292-shDUOX1 cells also demonstrated markedly increased ability of anchorage-independent growth on soft agar (Figure 1f). These findings were consistent across the three separate H292-shDUOX1 clones. Together, these findings suggest that DUOX1 suppression can initiate the process of EMT.

Analysis of various molecular features characteristic of EMT^33^ revealed that H292-shDUOX1 cells showed markedly reduced expression of E-cadherin, and upregulation of the mesenchymal markers vimentin, collagen and smooth muscle actin, compared with corresponding controls (Figure 1g; Supplementary Figure S1B). Moreover, H292-shDUOX1 cells also displayed constitutive activation of Akt, an important common hallmark of EMT^34, 35^ (Supplementary Figure S1B), as well as markedly reduced levels of miR-200b, miR-200c, and miR-205, and enhanced mRNA expression of EMT-promoting transcription factors ZEB1 and ZEB2 (Figure 1h), additional common molecular features of EMT.^36, 37^ To confirm the association of DUOX1 silencing with EMT in multiple epithelial cell lines, we performed similar analyses after repetitive siRNA-mediated DUOX1 silencing over a period of 9 days in both H292 cells and the non-cancer bronchial epithelial cell line HBE1, which revealed a similar gradual loss of E-cadherin and induction of mesenchymal markers in both cell models (Supplementary Figures S2A and B). As DUOX1 silencing also resulted in reduced expression of the DUOX1 maturation factor DUOXA1, we determined whether silencing of DUOXA1 itself was able to cause features of EMT. However, similar siRNA targeted suppression of DUOXA1 did not induce loss of E-cadherin, although it did enhance vimentin expression (Supplementary Figure S2C). Finally, induction of EMT features by DUOX1 silencing could be reversed by treatment with the DNA methyltransferase inhibitor 5-aza-deoxycytidine (aza), suggesting that DUOX1 suppression promotes EMT by an epigenetic mechanism (Supplementary Figure S3). Collectively, these findings indicate that loss of DUOX1 expression in lung epithelial cells directly induces loss of epithelial characteristics and promotes development of EMT.

### DUOX1 silencing in H292 cells is associated with enhanced resistance to EGFR tyrosine kinase inhibition

**Figure 2:**
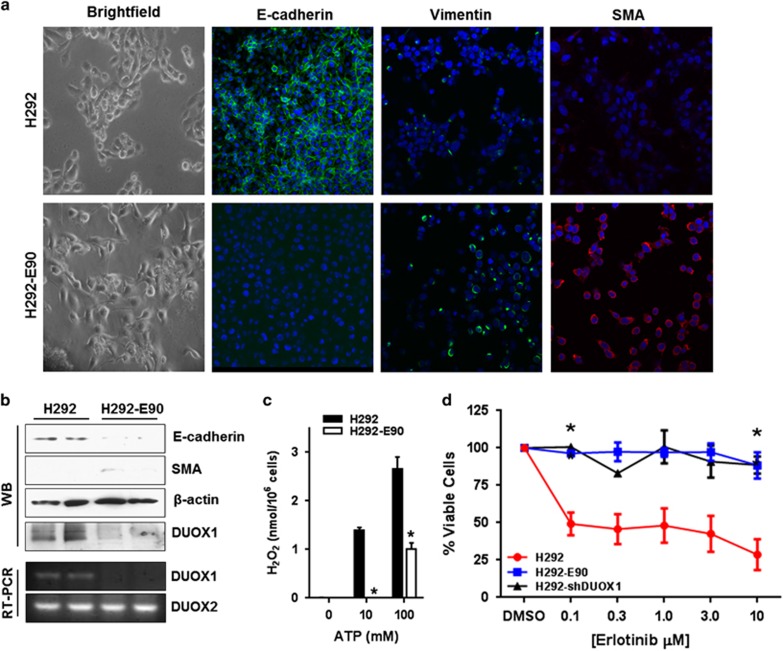
DUOX1 silencing is associated with acquired resistance to the EGFR TKI, erlotinib. (**a**) Brightfield and fluorescent confocal imaging of host cell line H292 and erlotinib-resistant H292 cell model (H292-E90) displaying features of EMT. (**b**) Protein and RNA analysis of EMT markers. (**c**) High-performance liquid chromatography H_2_O_2_ measurements of ATP-stimulated H292 and H292-E90 cell lines. (**d**) Percentage of viable cells post treatment of chemotherapeutic, erlotinib. *P<0.05 and **P<0.01 were calculated by one-way analysis of variance or Student's *t*-test.

The process of EMT has been associated with acquired resistance of lung cancers to chemotherapeutic drugs and to TKI of the EGFR.^38, 39^ Because of the close relationship between DUOX1 and EGFR-dependent signaling,^7, 8^ we explored the association between DUOX1 silencing, EMT and sensitivity to the EGFR TKI erlotinib. First, we cultured H292 cells for prolonged periods of time in the presence of erlotinib to select sub-populations with acquired erlotinib resistance (H292-E90). Consistent with earlier reports,^30^ thus-cultured erlotinib-resistant cells displayed morphological and molecular features of EMT (Figures 2a and b), which were partly reversible by treatment with aza (Supplementary Figure S2). Importantly, compared with normal H292 cells, H292-E90 cells also displayed marked loss of DUOX1 protein and mRNA expression (Figure 2b), and loss of functional DUOX1 activity (Figure 2c), further supporting a close association between DUOX1 silencing and EMT. Also, while normal H292 cells showed impaired growth in the presence of erlotinib, both H292-E90 and H292-shDUOX1 cells displayed marked resistance to erlotinib up to 10 μM (Figure 2d), indicating that DUOX1 suppression may be an important determinant of acquired erlotinib resistance in lung cancers. Importantly, to rule out the possibility that enhanced erlotinib resistance may be related to acquired activating mutations in EGFR in our engineered cell lines, we verified by DNA sequencing that neither H292-E90 and H292-shDUOX1 cells contained the following detectable EGFR point mutations at exon 19 D761Y, V769L and N771T or exon 20 T790M or any insertion mutations on exon 19 (D770-N771) (Supplementary Figure S4).

### DUOX1 silencing in H292 cells leads to enhanced CSC characteristics

**Figure 3:**
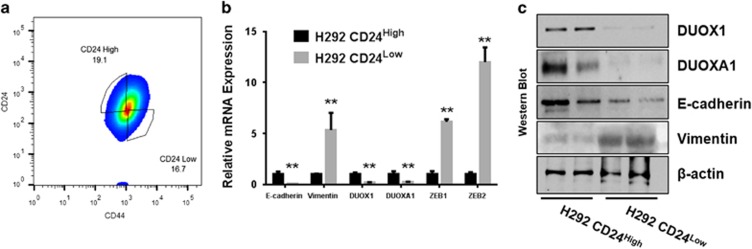
EMT features and loss of DUOX1 in the H292 CD24^low^ sub-population. (**a**) FACS gating strategy displaying H292 cell sub-populations were sorted based on their expression of surface antigen CD24/CD44. (**b**) Quantitative real-time PCR and western blot (**c**) results displaying CD24^low^ H292 sub-populations display features of EMT and marked DUOX1 suppression. *P<0.05 and **P<0.01 were calculated by Student's *t*-test.

**Figure 4:**
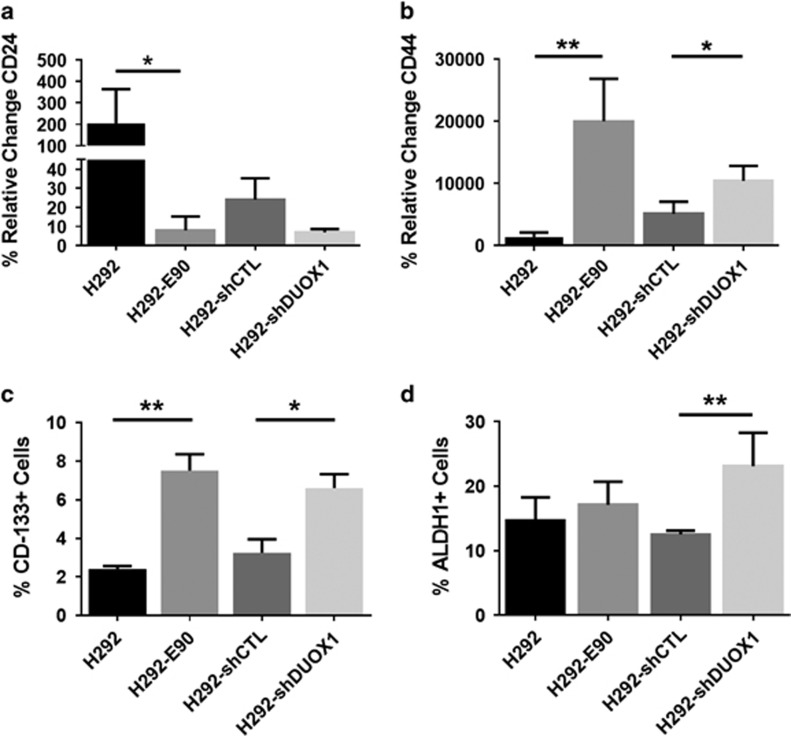
DUOX1 silencing enhances proportions of EMT-like CD24^low^/CD44^high^ cell populations and elevates expression of CSC markers CD-133 and ALDH1. (**a**, **b**) FACS results of relative percent change of surface antigen CD24 and CD44 (left/right, respectively) vs their unstained controls. (**c**) FACS results of %CD-133+ cells as compared with their individual controls. (**d**) FACS results of %ALDH1+ cells as compared with their controls. *P<0.05 and **P<0.01 were calculated by Student's *t*-test.

One characteristic feature of EMT is altered expression of cell surface markers such as CD44 and CD24, typically involving increased CD44 expression and reduced CD24 expression.^30, 40^ As established cancer cell lines are known to display heterogeneity with respect to CD44/CD24 expression, and contain CD44^high^/CD24^low^ sub-populations that are typically more mesenchymal in nature,^30, 40^ we used fluorescence-activated cell sorting (FACS) high-speed cell sorting to isolate CD24^low^/CD44^high^ sub-populations from H292 cells (Figure 3a; Supplementary Figure S5A) and confirmed that this sub-population demonstrated enhanced EMT characteristics compared with CD24^high^/CD44^low^ cells. Moreover, CD24^low^/CD44^high^ H292 sub-populations demonstrated marked suppression of DUOX1 mRNA and protein (Figures 3b and c; Supplementary Figure S5B), confirming the close relationship between loss of DUOX1 expression and EMT features. Conversely, analysis of H292-shDUOX1 cells as well as H292-E90 cells for mRNA levels and cell surface expression of CD24 and CD44 revealed that both H292-shDUOX1 and H292-E90 cells displayed reduced mRNA expression and cell surface levels of CD24, and enhanced mRNA and surface expression of CD44, compared with corresponding controls (Figures 4a and b; Supplementary Figure S5C), consistent with a shift toward a more mesenchymal phenotype in both cases.

The process of EMT is strongly associated with increased characteristics of CSCs, and CD24^low^/CD44^high^ sub-populations are thought to display increased CSC features,^41^ suggesting that DUOX1 silencing in lung cancer may also be associated with increased CSC characteristics. To address this, H292-E90 cells and H292-shDUOX1 cells were analyzed for the expression of two commonly accepted lung CSC markers, CD133 and ALDH1,^41, 42, 43^ using flow cytometry (Supplementary Figure S6). Indeed, both H292-shDUOX1 and H292-E90 cells were found to contain significantly increased populations of CD133+ cells compared with corresponding controls (6.61±0.72% vs 3.25±0.70% and 7.52±0.85% vs 2.41±0.16%, respectively) (Figure 4c). Similarly, H292-shDUOX1 cells also displayed an increased fraction of ALDH1+ cells compared with H292-shCTL cells (23.1±5.2% vs 12.5±0.6%), although no significant increase was observed in H292-E90 cells (Figure 4d). Finally, culturing of FACS-sorted CD133+ sub-populations in ultra-low attachment plates in stem cell media revealed enhanced growth of CD133+ cells from H292-shDUOX1 or H292-E90 cells compared with their control counterparts (Supplementary Figure S7). Together, these findings demonstrate that DUOX1 silencing in lung cancer is associated with increased CSC properties.

### DUOX1 overexpression reverses EMT features

**Figure 5:**
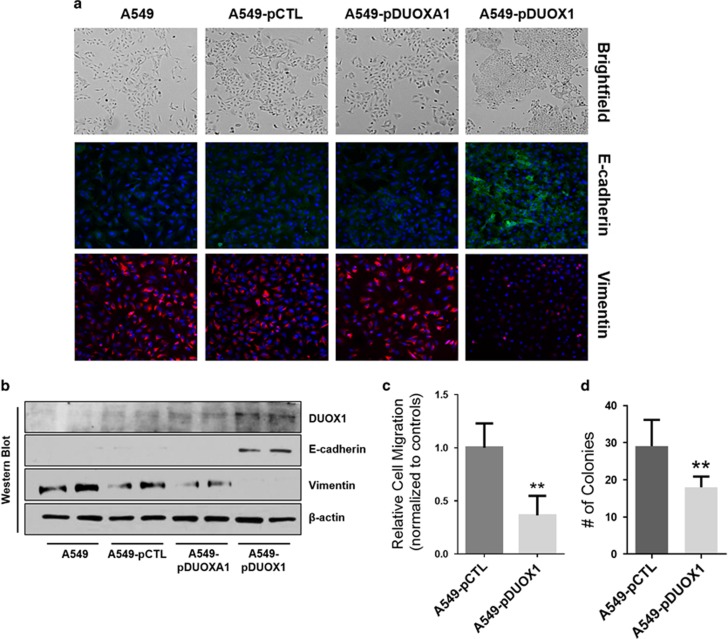
Overexpression of DUOX1 in A549 cells promotes epithelial signatures. (**a**) Brightfield microscopy reveals DUOX1-overexpressing cells display an epithelial cell morphology. Confocal imaging of epithelial marker E-cadherin and mesenchymal protein vimentin. (**b**) Western blot analysis shows DUOX1 overexpression promotes features of the mesenchymal-to-epithelial transition. (**c**) Donut cell migration assay displaying slower migration in DUOX1-overexpressing cells. (**d**) DUOX1 overexpression inhibits colony formation on soft agar. **P<0.01 was calculated by Student's *t*-test.

Although our results indicate that DUOX1 silencing in lung cancer cells promotes EMT and CSC features, the question remains whether these features can also be reversed by DUOX1 overexpression. To address this, we used the alveolar adenocarcinoma cell line A549, which displays low DUOX1 and E-cadherin expression and high levels of vimentin, and transfected them with DUOX1 cDNA (A549-pDUOX1; Supplementary Figure S8). As DUOX1 silencing was also associated with suppression of its maturation factor DUOXA1 (Supplementary Figure S2A), we similarly transfected A549 cells with DUOXA1 cDNA (A549-pDUOXA1) for comparison. Remarkably, compared with control A549 cells or A549-pDUOXA1 cells, A549-pDUOX1 cells displayed enhanced epithelial morphology with tight cell–cell contacts and increased cobblestone appearance (Figure 5a). Accordingly, DUOX1 overexpression enhanced protein expression of E-cadherin and suppressed expression of the mesenchymal marker vimentin, as visualized by immunofluorescence and western blot analysis (Figures 5a and b). DUOX1 overexpression in A549 cells also enhanced E-cadherin and reduced vimentin mRNA expression (Supplementary Figure S9). Overexpression of DUOX1, but not DUOXA1, also inhibited migratory capacity of A549 cells in a donut migration assay (Figure 5c; Supplementary Figure S9E) and in a scratch wound assay (Supplementary Figure S8C). Finally, DUOX1 overexpression in A549 cells significantly suppressed their ability to form colonies in soft agar (Figure 5d). Similar reversal of EMT features were observed after overexpression of DUOX1, but not DUOXA1, in the metastatic lung cancer cell line NCI-H187 (Supplementary Figure S10) in which DUOX1 is also silenced (Supplementary Figure S1). Collectively, these findings indicate that DUOX1 overexpression in lung cancer cells can indeed reverse molecular and functional features of EMT.

### DUOX silencing promotes tumor invasiveness

**Figure 6:**
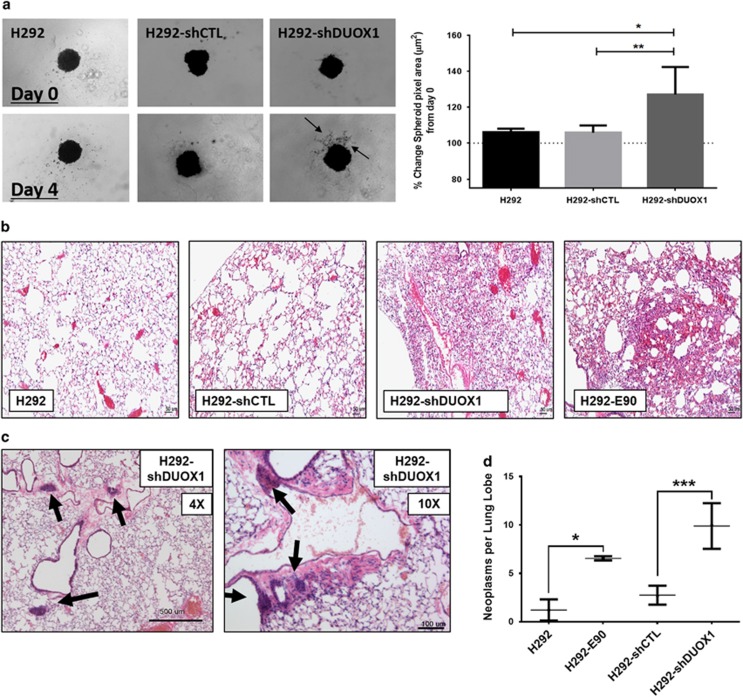
DUOX1 silencing displays enhanced invasive properties. (**a**) H292-shDUOX1 cell spheroids display invasion into surrounding extracellular matrix (ECM)-like matrix after 4 days, arrows display cellular invasion into mock ECM gel matrix. Right panel shows average percent change in pixel area quantified over the course of multiple experiments. *P<0.05, **P<0.01. (**b**) H&E-stained lung sections of H292-shDUOX1- or H292-E90-injected mice present significantly altered lung architecture. (**c**) Localized areas of neoplasias or small engrafted tumors in H292-shDUOX1-injected mouse lungs. (**d**) Quantification of counted lung neoplasias >120 μM in diameter for animals injected with various cell models. *P<0.05 and ***P<0.001 were calculated by Student's *t*-test.

As the process of EMT is strongly linked with increased invasive or metastatic potential of lung cancer cells,^33^ we evaluated whether DUOX1 silencing was associated with increased invasive/metastatic behavior. First, H292-shDUOX1 cells and corresponding controls were evaluated in a spheroid invasion assay, which indeed demonstrated significantly increased invasiveness of H292-shDUOX1 cells into the surrounding extracellular matrix, compared with H292-shCTL cells or untransfected control H292 cells (Figure 6a). To address *in vivo* invasiveness, we injected three clones of H292-shDUOX1 cells, as well as H292-E90 and corresponding control cells, into the tail vein of male CD-1 nude mice and collected major organs after 12 weeks for analysis. Indeed, lung tissues of mice injected with H292-shDUOX1 cells or the EMT cell line H292-E90 displayed altered lung architecture with hypercellularity (Figure 6b) and regions of atypical hyperplasia or small localized neoplasias (Figure 6c; lower panels). No significant abnormalities were observed in livers or spleens (not shown). To evaluate quantitative differences in neoplasias or metastatic lesions within the lung, we sectioned the entire left lung lobe of each animal and examined series of sections separated by about 50 μm for the presence of neoplasias with diameter >120 μm (Supplementary Figure S11). Total numbers of counted neoplasias are summarized in Figure 6d, and indicate that injection of H292-E90 or H292-shDUOX1 (average of all three H292-shDUOX1 clones) resulted in significantly greater numbers of neoplasias compared with their respective controls. Because our histological analysis does not identify the cellular origin of these lung lesions, invasion or engraftment of injected human tumor cells was also evaluated by quantitative PCR analysis of human ALU (hALU) repeat sequences, a DNA element not found in murine tissues. Indeed, mouse lung tissues with altered lung architecture and regions of hyperplasia also contained enhanced levels of hALU DNA (Supplementary Table S1). Although injection of normal H292 or H292-shCTL cells did not induce significant increases in hALU DNA in mouse lungs compared with naive mice (*n*=3), >5-fold increases in hALU DNA were detected in lung tissues after injection of H292-shDUOX1 cells in four out of seven cases, and after injection of H292-E90 (two out of three cases). Overall, these findings indicate that DUOX1 silencing in H292 lung cancer cells significantly enhances invasiveness and metastatic potential.

## Discussion

The main finding of the present studies is that they establish the potential significance of frequent observations of epigenetic silencing of the NADPH oxidase DUOX1 in lung cancer and in other epithelial-derived cancers,^18, 20, 21, 44^ and indicate that DUOX1 silencing is causally linked with the development or progression of EMT, and promotes invasive properties, as well as CSC features and resistance to EGFR TKIs. Our findings may be highly relevant for lung cancer, as the process of EMT is a critical determinant of tumor invasiveness and metastatic potential, and has been frequently linked with poor prognosis.^29, 33^ Furthermore, associations of DUOX1 silencing with increased CSC properties and resistance to EGFR inhibitors have important implications for anticancer therapies and suggest that development of approaches aimed at preventing or reversing DUOX1 silencing may be clinically useful in targeting invasive or metastatic lung cancer. Moreover, although EGFR inhibitors are typically not recommended for use in treatment of lung cancers expressing wild-type EGFR, some patients with wild-type EGFR may also benefit, and identification of biomarkers to identify such patients would be desirable.^45^ In this regard, our studies suggest that lung cancers with wild-type EGFR and positive DUOX1 expression may benefit for EGFR inhibitor therapy. The clinical relevance of DUOX1 suppression in lung cancer is also supported by a recent study of patients with hepatocellular carcinoma, which indicated that higher DUOX1 expression strongly correlated with disease-free survival.^44^ However, as our various observations were based on experimental cell models, the clinical relevance of our findings with respect to, for example, tumor stage or progression is still unclear.

Our present findings appear to contrast previous findings by us and others,^6, 7^ which indicated that inhibition of DUOX1 in epithelial cells attenuates cell migration in the context of epithelial wound injury responses, which involve the activation of DUOX1 by wound-derived signals such as ATP. The present findings, however, highlight a different scenario related to more gradual changes in epithelial biology and gene expression due to persistent loss of DUOX1, which leads to cell transformation with enhanced intrinsic cell motility, even though cells are less responsive to ATP-mediated wound responses.^7^

In addition to silencing of DUOX1, lung cancers were also found to display silencing of DUOXA1,^18^ a maturation factor for DUOX1, which aids in its trafficking to the plasma membrane to function as a competent NADPH oxidase.^2^ Indeed, our findings indicate that RNAi-dependent silencing of DUOX1 also leads to suppression of DUOXA1 (Supplementary Figure S3A), which might contribute to observed EMT phenomena (for example, Figure 3). Intriguingly, DUOXA1 was originally described as Numb interacting protein (NIP). Numb is a negative regulator of the Notch signaling pathway, which has an important role in airway basal cell differentiation.^46^ Uncontrolled Notch signaling has been associated with metastatic and aggressive epithelial-derived carcinomas, and nearly half of non-small-cell lung cancers display elevated Notch expression/signaling, often associated with suppressed Numb expression.^47^ DUOXA1/NIP expression often mimics the expression status of Numb and loss of Numb has been associated with disruption of the epithelial phenotype.^48^ In addition to regulating Numb, NIP/DUOXA1 also has an important role in, for example, myogenesis, with overexpression of NIP/DUOXA1 inhibiting differentiation and promoting apoptosis through enhanced DUOX1-dependent ROS production.^49^ To address the potential involvement of DUOXA1, we used either direct siRNA silencing or overexpression of DUOXA1, which did not significantly affect EMT (Supplementary Figure S2C; Figure 5). Therefore, although some molecular alterations associated with DUOX1 silencing may have resulted from indirect changes in, for example, Notch/Numb status due to suppression of DUOXA1, the observed effects on EMT appear to be primarily due to loss of DUOX1 itself.

The mechanism(s) by which DUOX1 silencing results in increased EMT still remain to be determined. It is plausible that a loss of DUOX1 activation and H_2_O_2_-dependent redox signaling might impact on regulation of Src activation and EGFR-dependent signaling pathways, based on recent studies linking DUOX1 and Src/EGFR activation in epithelial wound responses.^4, 7, 50^ Indeed, expression and activation of Src and EGFR are often increased in lung cancer and form a common therapeutic target in many lung cancers,^9, 11^ and acquired resistance to EGFR TKIs may be associated with increased nuclear localization of EGFR.^51, 52^ However, our studies did not indicate significant differences in overall ROS production in either DUOX1-deficient H292 cells^7^ or DUOX1-overexpressing A549 cells under basal conditions (Supplementary Figure S8C), unless DUOX1 was activated by external stimuli such as extracellular ATP. Although we cannot rule out a potential impact of basal DUOX1-derived H_2_O_2_ production in determining cellular outcomes, it is also possible that DUOX1 expression may control cell fate by redox-independent mechanisms, for example, by acting as a scaffold for EGFR or Src, similar to the recently described role of AKAP12.^53^ In addition, it is also plausible that altered DUOX1 status affects cellular redox-dependent mechanisms by more indirect effects, for example, by altered expression of other NOX isozymes. However, although DUOX1 loss or overexpression resulted in some modest alterations in mRNA expression of NOX1, 2 or 4 (Supplementary Figure S12), we did not observe consistent alterations that would point to a major role of other NOX isoforms in our observed outcomes. Future mechanistic studies will be required to address these various issues.

In summary, our findings indicate that DUOX1 silencing, a common finding in epithelial cancers, including lung cancer,^12, 20, 21^ may be a strong determinant of increased invasive or metastatic potential, as well as resistance to conventional anticancer therapies. At this stage, it is unknown how DUOX1 silencing relates to different types of lung cancer or with specific mutations in EGFR, KRAS and so on. Also, our findings appear to contrast with recent findings indicating a role for DUOX1-derived H_2_O_2_ as a cause of oxidative DNA damage and genomic instability in response to ionizing radiation of the thyroid, as a potential mechanism of development of thyroid cancer.^13^ However, it is plausible initiation of lung carcinogenesis by oxidative events related to DUOX1 may be followed by subsequent epigenetic DUOX1 silencing in established tumors, which in turn may serve to promote invasiveness or metastatic potential. Also, the observed association between DUOX1 expression and sensitivity to EGFR TKI may suggest that positive DUOX1 expression status in lung cancers may be suitable for therapeutic management with EGFR TKI, even if they express wild-type EGFR. Future studies are warranted to further decipher the mechanism(s) involved in epithelial DUOX1 expression and its relation to epithelial phenotype or development of EMT, which would allow for alternative approaches to therapeutically control DUOX1 expression as a potential treatment strategy in established lung cancer.

## Materials and methods

### Cell models and transfections

NCI-H292 cells, a human pulmonary mucoepidermoid carcinoma cell line (American Type Culture Collection; ATCC), were propagated in RPMI 1640 medium with 10% fetal bovine serum/5% penicillin–streptomycin. DUOX1-deficient H292 cells (H292-shDUOX1) and corresponding vector control cells (H292-shCTL) were generated and maintained as previously described.^7^ Three separate H292-shDUOX1 clones were expanded and utilized in these studies. Erlotinib-resistant sub-clones of H292 cells were generated essentially as described by Yao *et al.*,^30^ by culture for 30 days in the presence of 5 μM erlotinib followed by 60 days in the presence of 10 μM erlotinib (LC Laboratories, Woburn, MA, USA). Selected resistant H292 cells (H292-E90) were maintained in the presence of 10 μM erlotinib and switched to regular growth media for assays. Alveolar adenocarcinoma cell line A549 cells (ATCC) were cultured according to ATCC guidelines, and grown to ~70% confluence in a 24-well dish (Costar, Corning, Corning, NY, USA) for transfection with 1 μg of cDNA clone of the following vectors: pCMV6-AC-GFP control vector (A549-pCTL) (PS100010, Origene, Rockville, MD, USA); pCMV6-DUOXA1-GFP (A549-pDUOXA1) (RG206754, Origene); or pCMV6-DUOX1-GFP (A549-pDUOX1) (RG223832, Origene). Cells were transfected 2 × over the span of 6 days and then cultured in the presence of 250 μg/ml neomycin (G418 sulfate) (Calbiochem, Millipore, Billerica, MA, USA) for 14 days. Positive transfection of DUOXA1 and/or DUOX1 in A549-pCTL/A549-pDUOXA1/A549-pDUOX1 cell lines was verified by western blot and qRT–PCR analysis.

Other cell lines used in this study include the following: NHBE (normal human bronchial epithelial cells) maintained as described previously;^3^ HBE1 (immortalized human bronchial epithelial cells) maintained as described previously;^3, 7^ BEAS2B (human bronchial epithelial cells); Calu-3 (human lung adenocarcinoma cells); NCI-H1975 (human lung adenocarcinoma cells); NCI-H1437 (human lung adenocarcinoma cells); NCI-H2170 (human lung squamous cell carcinoma cells); NCI-H82 (human lung carcinoma cells); NCI-H460 (human lung carcinoma cells); and NCI-H187 (human lung retinoblastoma cells). Unless indicated otherwise, all cell lines were obtained from ATCC and maintained according to established ATCC protocols, as well as surveyed for mycoplasma contamination.

### Trans-epithelial electrical resistance

Cells were seeded in transwell culture inserts and grown to confluency. Trans-epithelial electrical resistance was measured using an EVOM epithelial voltohmmeter (World Precision Instruments, Sarasota, FL, USA). Trans-epithelial electrical resistance values were corrected for background resistance provided by the insert and/or the media. Data are represented as mean±s.d. **P<0.01 were calculated by two-tailed Student's *t*-test. (*n*=6; replicated 2 ×).

### Anchorage-independent growth

Six-well culture plates were coated with 2 ml of a 50/50 mixture of 1% agarose and 2 × growth medium, and appropriate cells were collected, resuspended in 50/50 mixtures of 0.67% agarose and 2 × complete medium and seeded onto agarose-coated culture plates and placed in a 37 °C incubator. After 4 weeks, plates were stained with a 0.005% crystal violet and imaged, and colonies were counted. Data are represented as mean±s.d. **P<0.01 were calculated by two-tailed Student's *t*-test. (*n*=6; replicated 2 ×).

### *In vitro* assays of cell migration and invasion

#### Scratch wound assay

A linear scratch (~2 mm) was generated in confluent cell monolayers in 24-well culture plates, and detached cells were removed with phosphate-buffered saline (PBS) and wound closure was monitored over a 24 h period. Wound areas were imaged and analyzed using NIH Image J software (National Institutes of Health, Bethesda, MD, USA) to determine % wound closure as an indicator of cell migration. Data are represented as mean±s.d. **P<0.01 were calculated by two-tailed Student's *t*-test. (*n*=6; replicated 2 ×).

#### Transwell cell migration assay

Cells were seeded in Boyden-like chambers containing 10 mm polycarbonate membrane inserts (8 μm pore size) (Nunc International, Penfield, NY, USA) that were pre-coated with 10 μg/ml fibronectin (Invitrogen). Following incubation for 24 h at 37 °C, non-migratory cells were removed from membrane with a cotton swab, and migrated cells were stained with crystal violet for 30 min and extracted in 100 μl of 0.2 M sodium acetate buffer (pH 4.5) for analysis of absorbance at 570 nM. Data are represented as mean±s.d. *P<0.05 were calculated by two-tailed Student's *t*-test. (*n*=6; replicated 2 ×).

#### Donut cell migration assay

Cells were seeded inside silicone circular gaskets at 10 000 cells per donut. Cells were maintained for ~24 h in complete cell medium to reach full confluency. Donuts were then removed and images were taken immediately following donut removal and 24 h later. Data were quantified using an ImageJ macro as described and provided previously.^32^ Data are represented as mean±s.d. *P<0.05 were calculated by two-tailed Student's *t*-test. (*n*=12; replicated 2 ×).

#### *In vitro* spheroid invasion assay

Invasive properties of the various cell lines were evaluated using a spheroid invasion assay (Cultrex Spheroid Invasion kit; Trevigen, Gaithersburg, MD, USA) according to the manufacturer's instructions. Images were taken on an inverted Olympus IX70 brightfield microscope and subsequently the images were analyzed using NIH Image J software as defined in Cultrex protocol. Data are represented as mean±s.d. *P<0.05; **P<0.01 were calculated by one-way analysis of variance (*n*=18; replicated 2 ×).

### Western blot analysis

Cells cultured in 24-well culture plates were lysed using 100 μl of 1 × western solubilization lysis buffer (1% Triton, 50 mM HEPES, 250 mM NaCl, 10% glycerol, 1.5 mM MgCl, 1 mM phenylmethylsulfonyl fluoride, 1 mM EGTA, 2 mM Na_3_VO_4_, 10 μg/ml aprotinin and 10 μg/ml leupeptin; pH 7.4) per well. Equal amounts of protein (20–25 μg; determined using bicinchoninic acid protein assay) were separated on Novex 10% Tris-Glycine gels (Life Technologies, Grand Island, NY, USA), transferred to nitrocellulose membranes and blotted using antibodies against the following: vimentin (#5741; 1:500; Cell Signaling, Danvers, MA, USA); Akt (#9272; 1:1000; Cell Signaling); p-Akt (#4060X; 1:1000; Cell Signaling); β-actin (#MA5-15739; 1:10  000; Invitrogen, Waltham, MA, USA); Collagen (#ab34710; 1:500; Abcam, Cambridge, MA, USA); E-cadherin (#3195; 1:1000; Cell Signaling); DUOX1 (1:1000; kindly provided by F. Miot^3, 54^); and DUOXA1 (#H00090527-B01P; 1:1000; Abnova, Jhongli, Taiwan), and detected using enhanced chemiluminescence (Pierce, Rockfort, IL, USA).

### Immunohistochemical analysis and morphological imaging

H292, H292-shCTL, H292-shDUOX1 and H292-E90 cells were seeded in eight-well glass chamber slides, fixed with 4% paraformaldehyde and permeabilized with 0.2% Triton X-100 in 1% bovine serum albumin (BSA) in PBS for 15 min. Slides were washed 2 × with 1% BSA/PBS solution, blocked with 10% goat serum for 1 h and then subjected to staining with antibodies against E-cadherin (1:100; Invitrogen), vimentin (1:100; Cell Signaling) or α-smooth muscle actin (1:100; Cell Signaling). Nuclei were counterstained with 4,6-diamidino-2-phenylindole (10 μg/ml in 1% BSA/PBS). Cells were washed 2 × with 1% BSA/PBS and mounted with glass coverslip. Images were taken on a Zeiss LSM 510 META laser scanning confocal microscope (Zeiss, Jena, Germany). Brightfield images were taken using a Zeiss Interskop phase contrast microscope interfaced with a digital camera (Zeiss).

### RNA interference

Cells were grown to ~70% confluence in 24-well plates and transfected with 0.1 μM siRNA targeted against DUOX1 mRNA (#117546, Ambion siRNA, Life Technologies; accession No. NM_017434.3/NM_175940.1), DUOXA1 mRNA (#90527, Dharmacon, GE, Lafayette, CO, USA; accession No. NM_144565.2) or 0.1 μM non-targeting control siRNA (Dharmacon, GE) in RPMI 1640 media (Gibco, Life Technologies), and incubated for 24 h at 37 °C. Media then replaced with complete media, and cells were incubated for an additional 48 h and then subjected to one or two successive similar transfections, and then collected (3, 6 or 9 days after initial transfection for extraction of miRNA, RNA or protein).

### Analysis of cell viability and proliferation

Cells were seeded at 1 × 10^4^ per well into a 96-well black flat bottom fluorescence plates (Nunc International, Rochester, NY, USA) and cultured in RPMI 1640 (no fetal bovine serum) in the presence of various doses of erlotinib (0.1–10 μM; LC Laboratories), by exchanging media±erlotinib daily for up to 3 days, after which cell viability was determined using ATP TiterGlo assay reagent (Promega, Madison, WI, USA) Data are represented as mean±s.d. *P<0.05; **P<0.01 were calculated by one-way analysis of variance (*n*=6; replicated 2 ×). Cell proliferation was analyzed by cell seeding at various densities and culture in full growth media for up to 72 h, using ATP TiterGlo reagent. Data are represented as mean±s.d. Data did not reach significance (*P*<0.05) as tested by a two-tailed Student's *t*-test (*n*=6; replicated 2 ×). Additional cell proliferation assays were performed by seeding cells at equal cell densities and collected daily for 7 days, and counted to assess proliferation. Data are represented as mean±s.d. Data did not reach significance (*P*<0.05) as tested by a two-tailed Student's *t*-test (*n*=6; replicated 2 ×).

### RNA extraction, PCR and quantitative real-time PCR

Total RNA was extracted with GeneJET RNA purification kit (Thermo Scientific, Waltham, MA, USA). For mature miRNA extraction, miRNeasy mini kit was utilized (Qiagen, Valencia, CA, USA). RNA extracts were reverse-transcribed and real-time PCR was performed using SYBR green quantitative PCR assays. All quantitative PCR primers were purchased from Sigma-Aldrich (St Louis, MO, USA) unless otherwise noted. Mature miRNA was reverse-transcribed utilizing miScript II RT Kit, and subsequent real-time PCR was performed with miScript SYBR Green PCR Kit and miScript Primer Assays (Qiagen). Data are represented as mean±s.d. *P<0.05; **P<0.01 were calculated by two-tailed Student's *t*-test or one-way analysis of variance (*n*=6; replicated 2 ×).

### Evaluation of CSC characteristics

CSC markers were evaluated by multi-color flow cytometric analysis (FACS) on a BD FACS LSRII (BD Biosciences, San Jose, CA, USA), after cell staining with 0.5 μg per 100 μl of anti-CD24-Pacific Blue (eBioscience, San Diego, CA, USA) and anti-CD44-Alexafluor 700 (Biolegend, San Diego, CA, USA), 0.5 μg per 100 μl of anti-human-CD133-AF700 (MACS, Auburn, CA, USA) or ALDEFLUOR reagent (0.5 μg per 100 μl; Stem Cell Technologies, Vancouver, BC, CA). Data are represented as mean±s.d. *P<0.05; **P<0.01 were calculated by two-tailed Student's *t*-test (*n*=6; replicated 2 ×). CD24^low^/CD24^high^ sub-populations of NCI-H292 cells were sorted utilizing anti-CD24-Pacific Blue antibody, using a BD FACSAria high-speed cell sorter (BD Biosciences). Similarly, CD133+ cells were sorted and collected in high fetal bovine serum (20%)-containing RPMI-1640 cell media (Gibco, Grand Island, NY) supplemented with 5% penicillin/streptomycin. Cells were centrifuged, washed 2 × with 1 × PBS, and resuspended in StemPro mesenchymal stem cell growth media supplemented with StemPro MSC SFM supplement (Gibco) and placed in ultra-low attachment plates (Corning) and monitored over the course of 7 days.

### *In vivo* tumor metastasis

Charles River CD1 nude male mice (8 weeks) were injected into the tail vein with 1 × 10^6^ of various cell lines (H292, H292-shCTL, H292-shDUOX1 (three separate clones) or H292-E90) in 1 × sterile PBS, at random. Mice were weighed twice weekly and monitored for overall health up until 12 weeks post injection, after which mice were killed and tissues collected. Lung sections were cut at a thickness of 5 μm and stained with hematoxylin and eosin (H&E) for visual observation of lung structure. To quantify total numbers of neoplasias, lung tissue blocks were cut at a thickness of 5 μm and stained for H&E, then tissue blocks were cut into 50 μm, and another 5 μm section was isolated for H&E staining. This process was repeated until the tissue block was exhausted. Each tissue section was examined and neoplasias >120 μm were counted. Neoplasia size was determined by measurements taken on Olympus BX-50 imaging software (Olympus, Center Valley, PA, USA). Neoplasm quantification was not performed under blinded conditions. Data are represented as mean±s.d. *P<0.05; ***P<0.001 were calculated by two-tailed Student's *t*-test (for H292, H292-E90 and H292-shCTL, *n*=3 each; for H292-shDUOX1, *n*=7). Genomic DNA from mouse lungs was isolated utilizing phenol–chloroform extraction method and 30 ng of DNA was further amplified by quantitative PCR for human ALU DNA sequences.^55^ All animal experiments were performed in compliance with IACUC regulations and guidelines.

### Analysis of DUOX1 activity

DUOX1 activity was evaluated by extracellular H_2_O_2_ production in response to cell stimulation with 100 μM ATP (Sigma-Aldrich), as described previously.^7^ Data are represented as mean±s.d. **P<0.01 were calculated by two-tailed Student's *t*-test (*n*=6; replicated 2 ×).

### Analysis of EGFR mutations

To determine potential acquisition of EGFR mutations in various engineered cell lines, cDNA was PCR amplified for EGFR exon 18 (5′-GCTGAGGTGACCCTTGTCTC-3′ (F), 5′-ACAGCTTGCAAGGACTCTGG-3′ (R)), EGFR exon 19 (5′-GCTGGTAACATCCACCCAGA-3′ (F), 5′-GAGAAAAGGTGGGCCTGAG-3′ (R)), EGFR exon 20 (5′-CCTCCTTCTGGCCACCATGCG-3′ (F), 5′-CATGTGAGGATCCTGGCTCC-3′ (R)) and EGFR exon 21 (5′-CGGATGCAGAGCTTCTTCCC-3′ (F), 5′-AGGCAGCCTGGTCCCTGGTG-3′ (R)), purified and prepared for Sanger sequencing utilizing the forward (F) primers listed above. Samples were analyzed on an ABI Prism 3130xl Genetic Analyzer (Thermo Scientific) at the University of Vermont DNA Analysis Core Facility.

### Statistical analysis

All experimental procedures are reported with ⩾4 individual replicates and replicated experimentally at a minimum of two times. All data are subjected to either students two-tailed *t*-test or a one way analysis of variance for the determination of statistical significance based on the set being analyzed. *P*-values <0.05 (*), *P*-values <0.01 (**). Correction for multiple comparisons were made using either Welch's correction or the Holm–Sidak method depending on the data set. All data were calculated in Microsoft Excel 2010, Graphpad prism 6 or NIH ImageJ.
