# Supplementary material for: DUOX1 silencing in lung cancer promotes EMT, cancer stem cell characteristics and invasive properties
Source: Oncogenesis. 2016 Oct 3;5(10):e261–. doi: 10.1038/oncsis.2016.61 (PMC5117847; doi:10.1038/oncsis.2016.61)
Supplement: Supplementary Figure [file oncsis201661x1.doc]

##### **Little et al., DUOX1 silencing in lung cancer promotes EMT, acquisition of cancer stem cell characteristics, and invasive properties**

**Supplementary Figures**

***
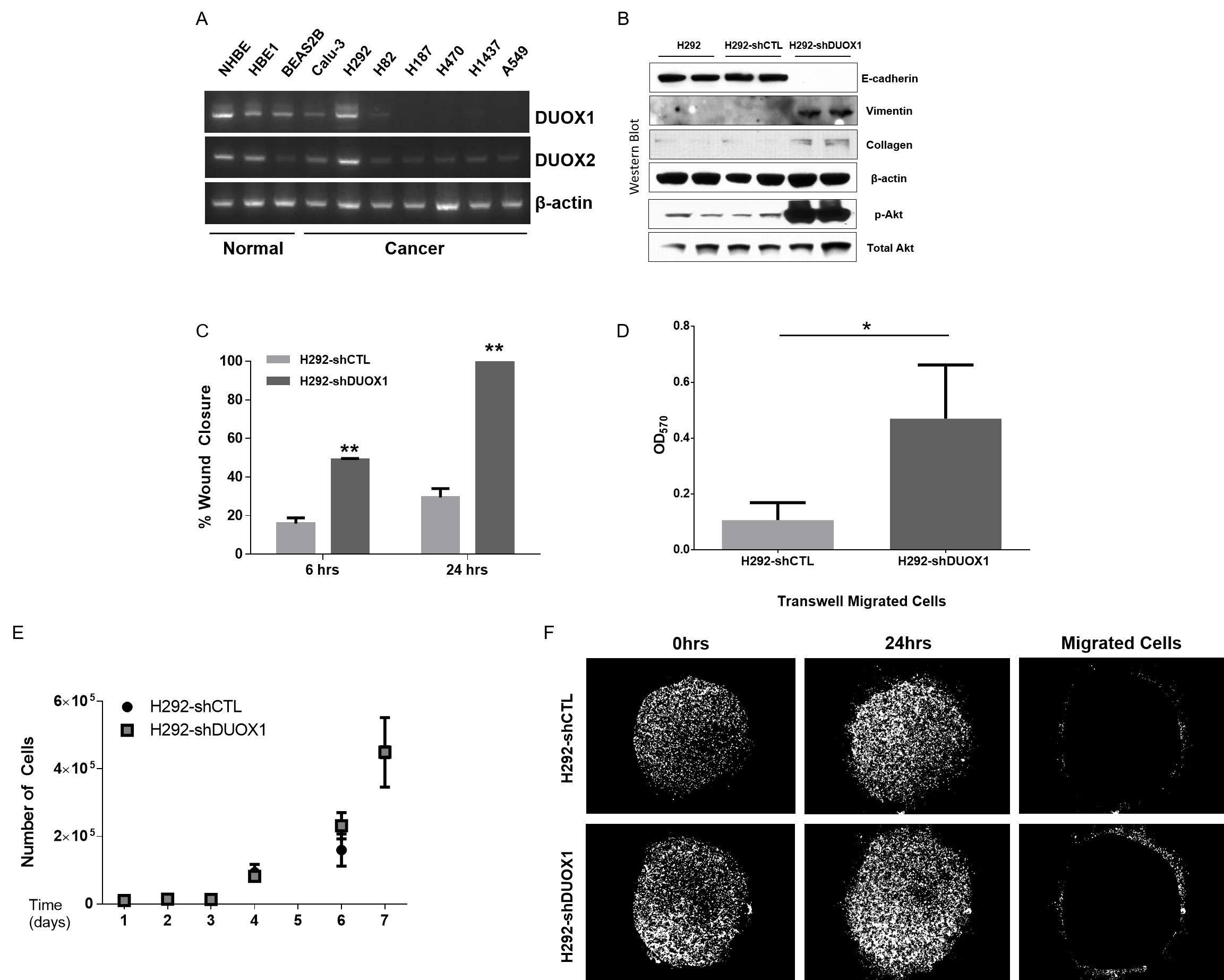
***

***Fig. S1.*** DUOX1 silencing promotes features of EMT. (A) Evaluation of DUOX1 and DUOX2 mRNA expression in normal airway epithelial cells and a panel of lung cancer cell lines. (B) Western blot analysis of molecular features of EMT in H292 cells after constitutive silencing of DUOX1 (H292-shDUOX1). (C,D) Analysis of cell migration of H292-shDUOX1 and corresponding controls in a scratch wound assay (n=6) (C) and in a Transwell migration assay (n=6) (D). (E) Evaluation of cell proliferation after cell seeding at 50,000/well (n=6). (F) Representative images from donut cell migration assay. Migrated cells (right most panel), represent total migrated cells 24hrs post removal of the silicon gasket (donut) (24hrs image – 0hrs image = migrated cells). Results are expressed as mean ± SD *p<0.05, **p<0.01 were calculated by Student’s t-test.


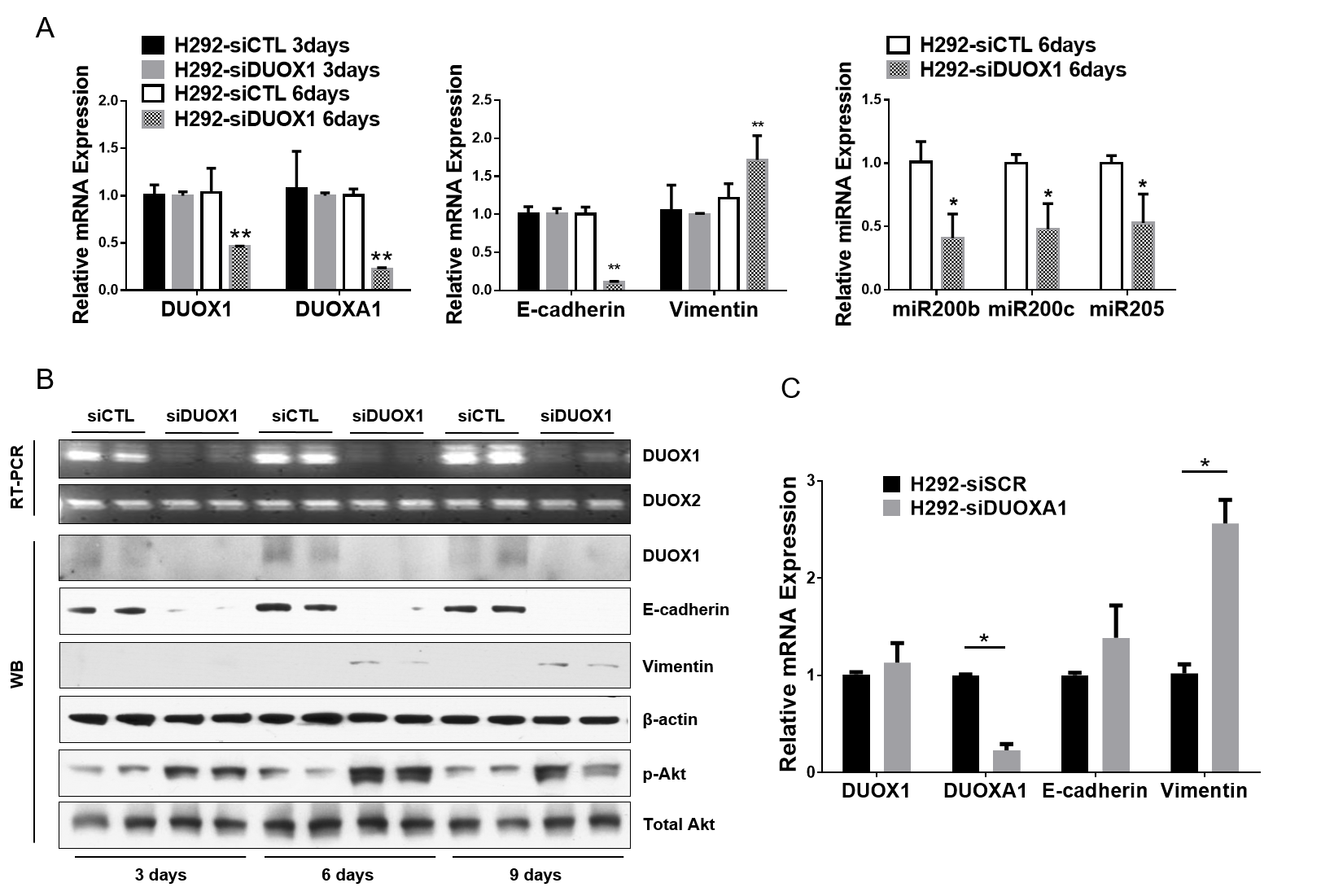


***Figure S2.*** Transient siRNA targeting of DUOX1 promotes features of EMT. (A) H292 cells were transfected with DUOX1-targeted siRNA or control siRNA 3X over the course of 9 days, and alterations in DUOX1, DUOXA1, E-cadherin, vimentin, or miR200 members were analyzed by RT-PCR (n=4 per group). (B) RT-PCR or Western blot analysis of DUOX1 and DUOX2 and EMT markers of after repeated siRNA-dependent silencing of DUOX1 in HBE1 cells . (C) H292 cells were transfected with DUOXA1 siRNA 3X over the course of 9 days, and DUOX1, DUOXA1, E-cadherin or vimentin were analyzed by qPCR (n=4 per group). Data are represented as mean ± SD *p<0.05, **p<0.01 were calculated by ANOVA or Student’s t-test.

***
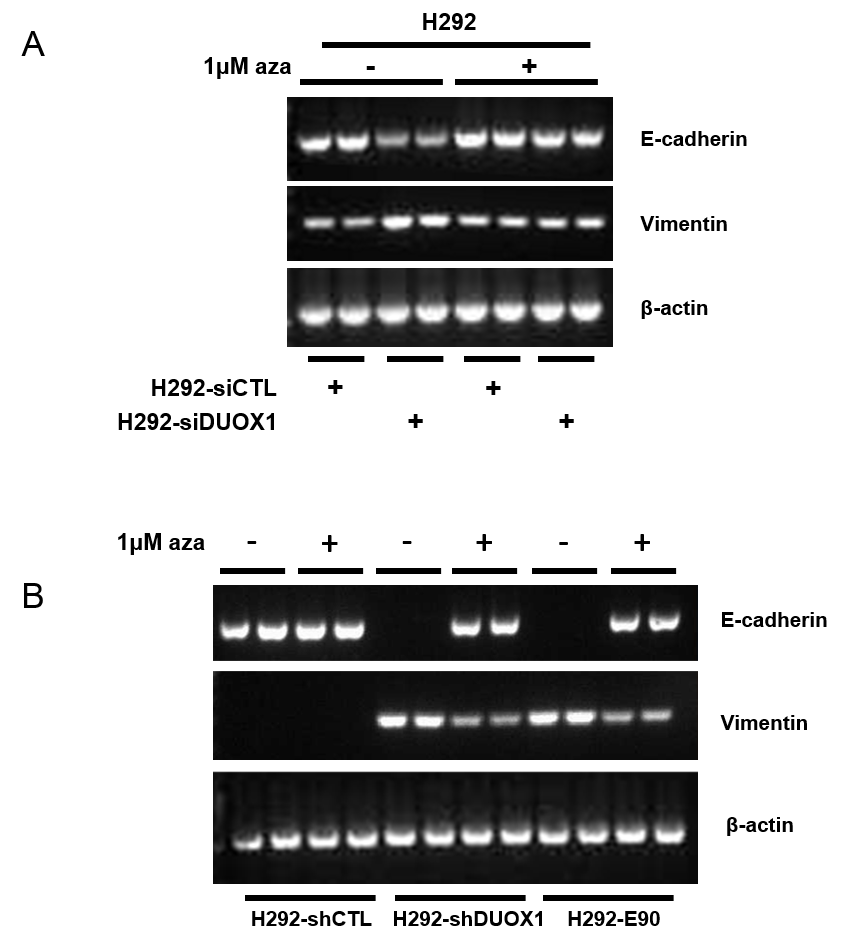
***

***Figure S3.*** DUOX1 silencing-induced EMT is mediated by epigenetic mechanisms. Control H292 cells (H292-shCTL), DUOX1-silenced cells (H292-shDUOX1) and erlotinib-resistant H292 cells (H292-E90) were incubated in the absence or presence of 5-azadoxycytidine (aza) for 4 days and mRNA levels of E-cadherin or vimentin were evaluated by RT-PCR.

***
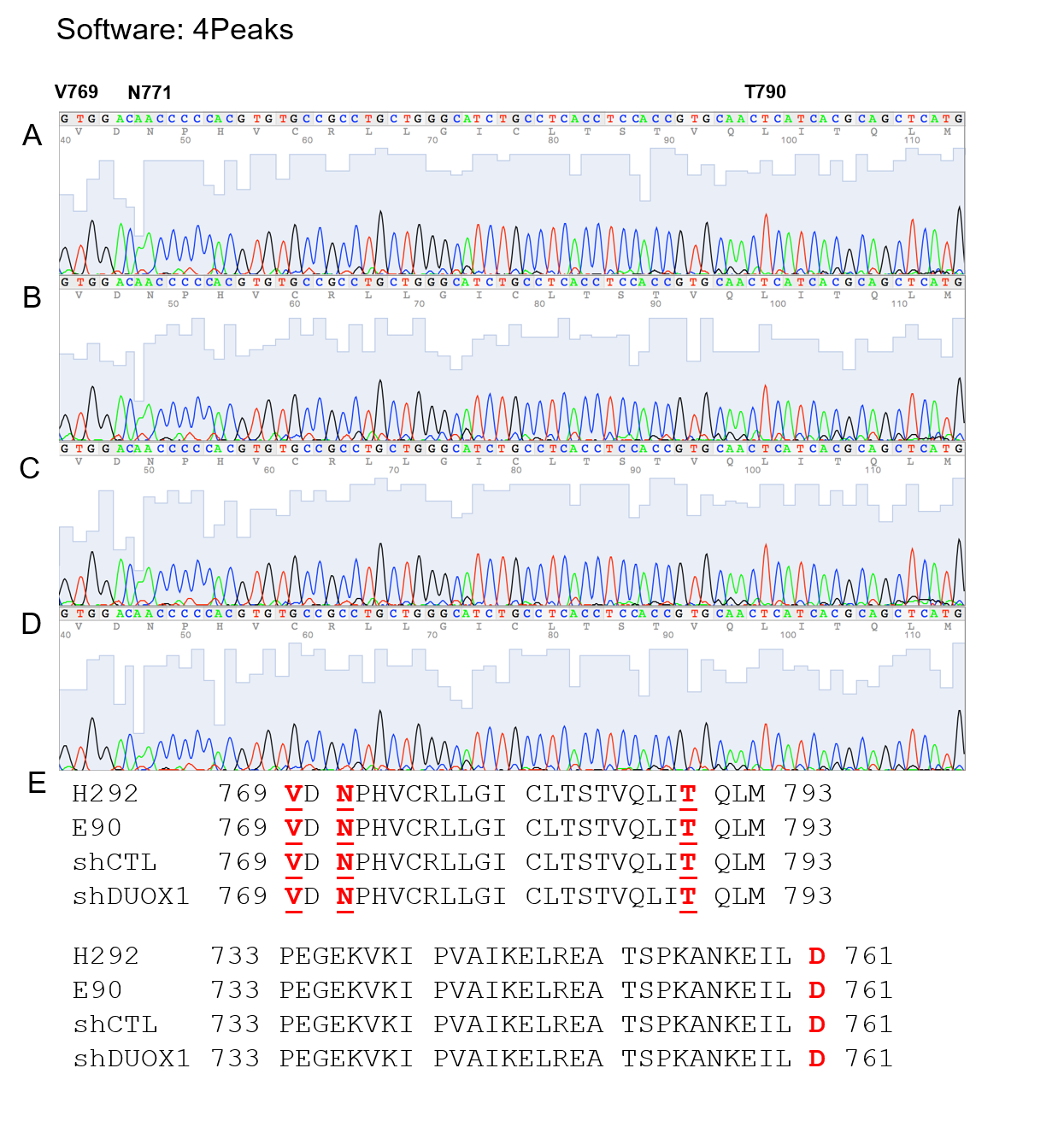
***

***Figure S4.*** Acquired resistance to erlotinib or DUOX1 silencing does not promote the acquisition of EGFR-TKI resistance point mutations. We confirmed by DNA Sanger sequencing that the host cell line H292 (A), erlotinib resistant H292-E90 cell model (B), H292-shCTL (C), or the H292-shDUOX1 (D) cell lines did not display point mutations V769L, N771T, D761Y, or T790M (E).

***
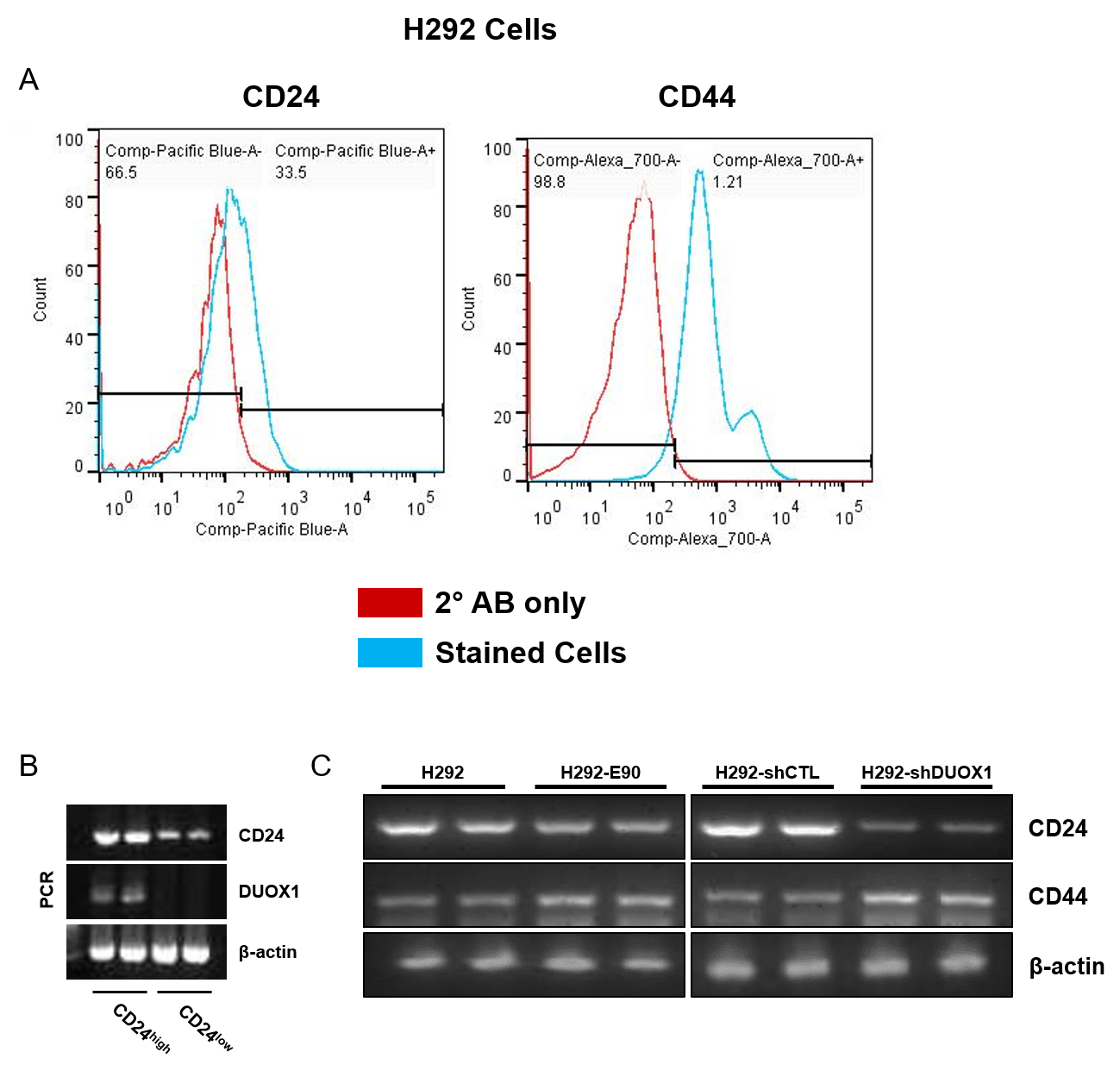
***

***Figure S5***. (A) FACS gating strategy for H292 cells against CD24 and CD44. (B) Characterization of FACS sorted CD24^high^ and CD24^low^ subpopulations of H292 cells for CD24 and DUOX1 mRNA expression by RT-PCR. (C) Analysis of CD24 and CD44 mRNA by RT-PCR in H292-E90 and H292-shDUOX1 compared to corresponding controls.

***
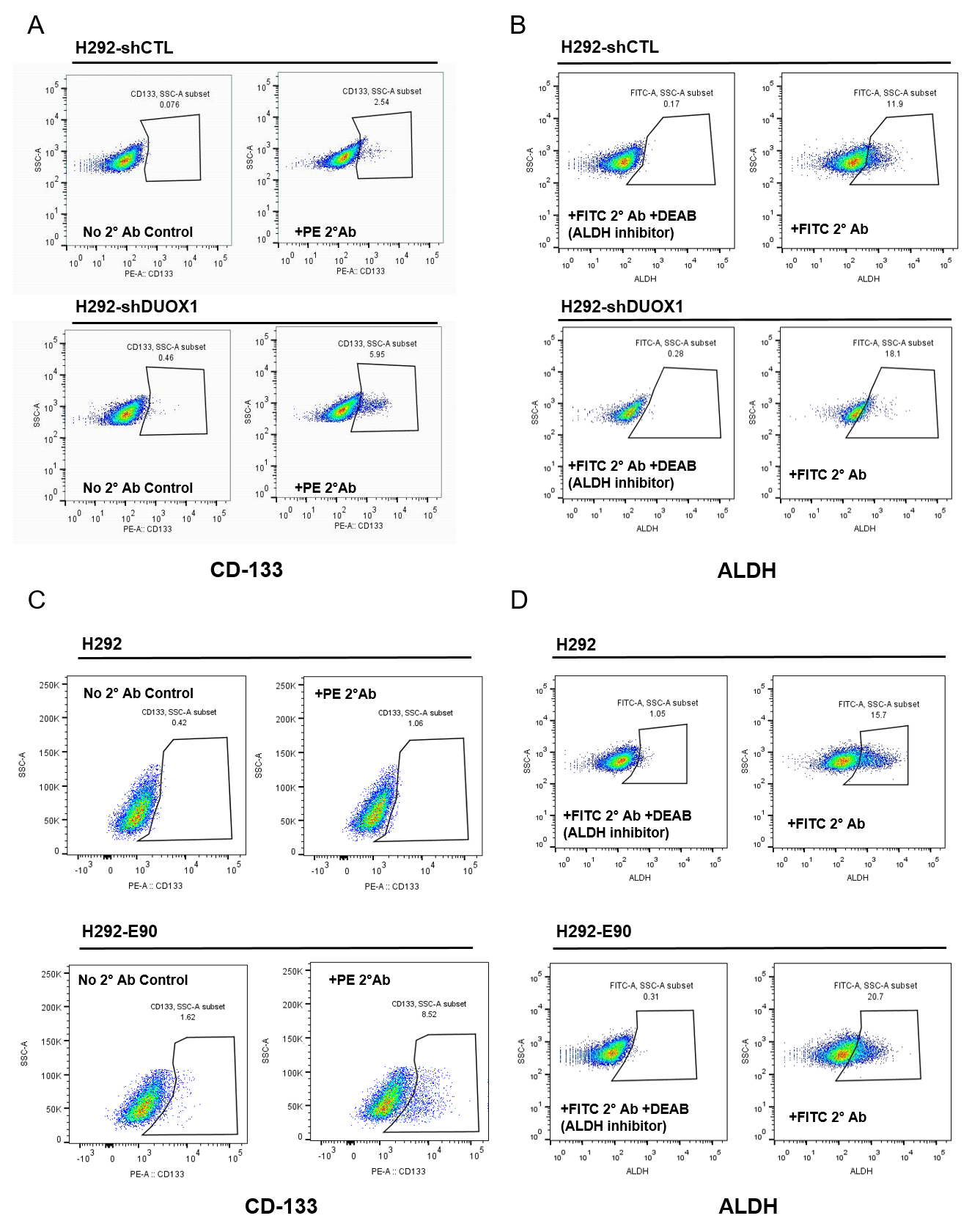
***

***Figure S6.*** FACS gating strategy for evaluation of surface expression of CD133 in H292-shCTL and H292-shDUOX1 cells (A) and in H292 and H292-E90 cells (C). (B,D) FACS gating strategy employed to monitor the expression of intracellular ALDH activity in the same cell models. Controls contain DEAB, an inhibitor of ALDH enzymatic activity.

***
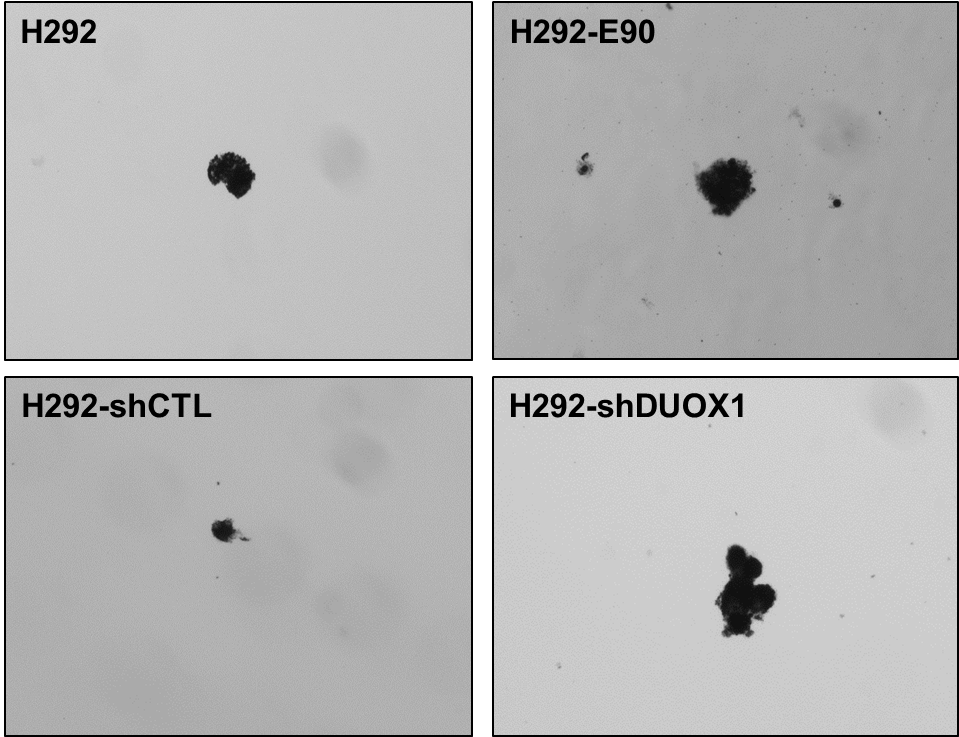
***

***Figure S7.*** Evaluation of CD133+ subpopulations of various cell lines for stem cell properties. CD133+ subpopulations were obtained by high speed FACS cell sorting and cultured in ultra-low attachment plates in mesenchymal stem cell growth media. Images were taken 7 days post cell sorting.

***
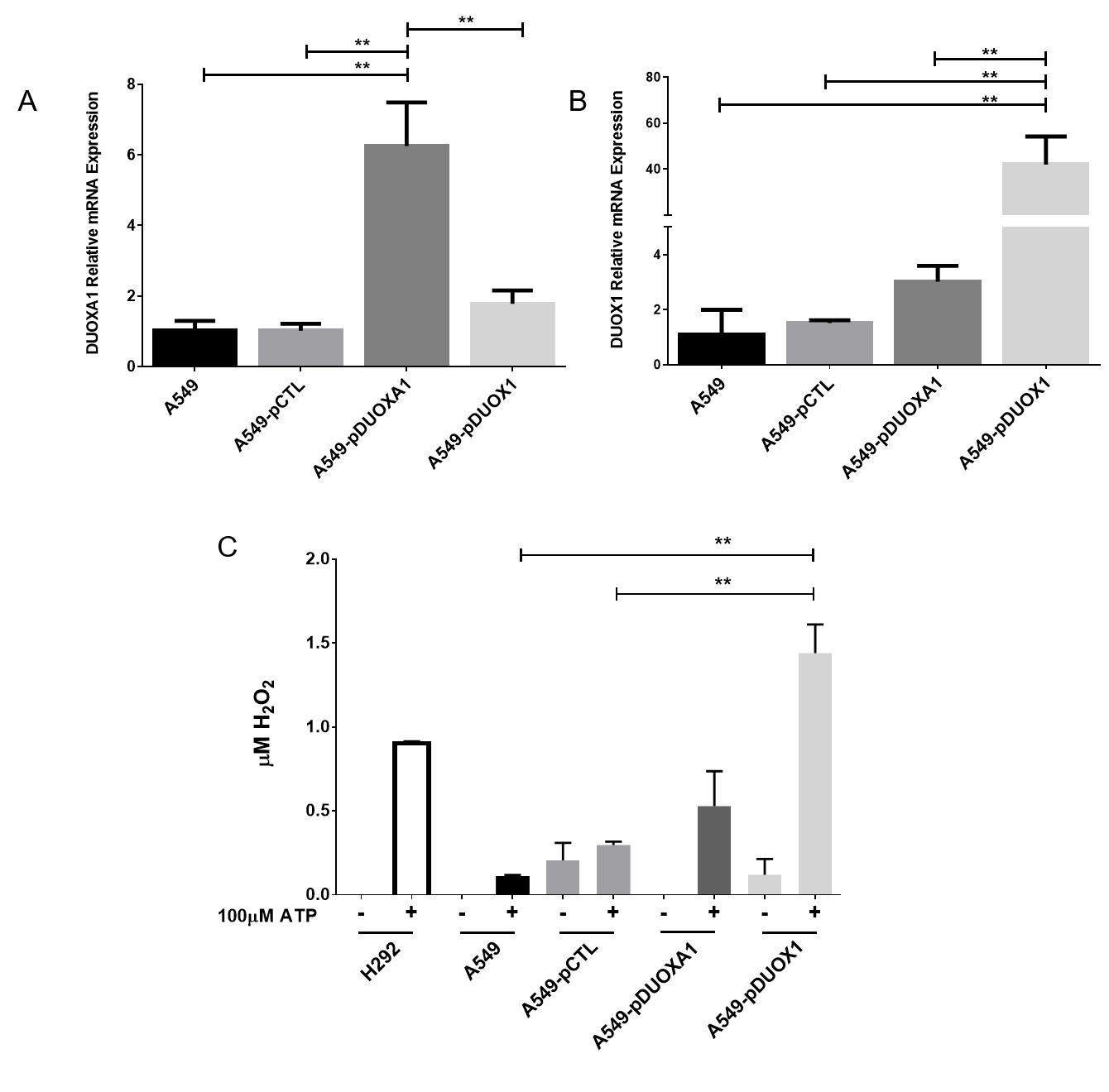
***

***Figure S8.*** Characterization of A549 cells transfected with DUOX1 or DUOXA1 cDNA, by qRT-PCR analysis of DUOXA1 mRNA (A) or DUOX1 mRNA (B) (n=4 per group), and by HPLC analysis of DUOX-dependent H_2_O_2_ production in response to cell stimulation with ATP (100 μM) (n=6). H292 cells were analyzed for comparison. Data are represented as mean ± SD *p<0.05, **p<0.01 were calculated by one-way ANOVA or Student’s t-test.


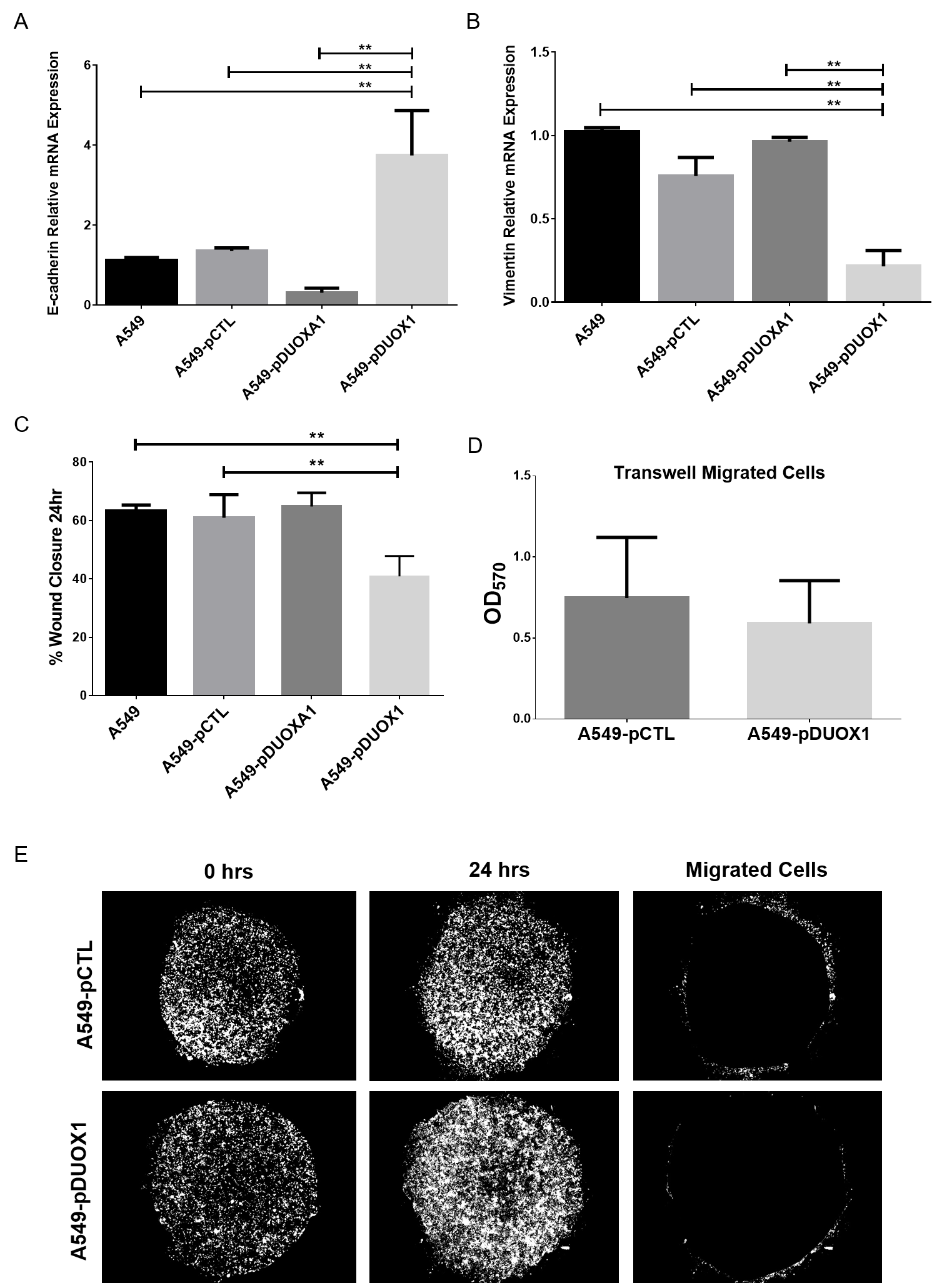


***Figure S9.*** Effect of overexpression DUOX1 or DUOXA1 on EMT features in A549 cells. Control or transfected A549 cells were analyzed for mRNA levels of the epithelial marker E-cadherin (A) and the mesenchymal protein vimentin (B) by qRT-PCR (n=4 per group). Control or transfected A549 cells (A549-pDUOX1) were analyzed for cell migration in a scratch wound assay (C) and in a Transwell migration assay (D). Results are expressed as mean ± SD (n=4). (E) Representative images from donut cell migration assay. Migrated cells (right most panel), represent total migrated cells 24hrs post removal of the silicon gasket (donut) (24hrs image – 0hrs image = migrated cells). *p<0.05, **p<0.01 were calculated by one-way ANOVA or Student’s t-test.

***Figure S10.*** Effect of DUOXA1 or DUOX1 overexpression on EMT features in H187 cells. Control or DUOX1/DUOXA1-transfected A549 cells were analyzed for mRNA expression levels of the epithelial marker E-cadherin and the mesenchymal protein vimentin by qRT-PCR. Results are expressed as mean ± SD (n=4) *p<0.05, **p<0.01 were calculated by one-way ANOVA.


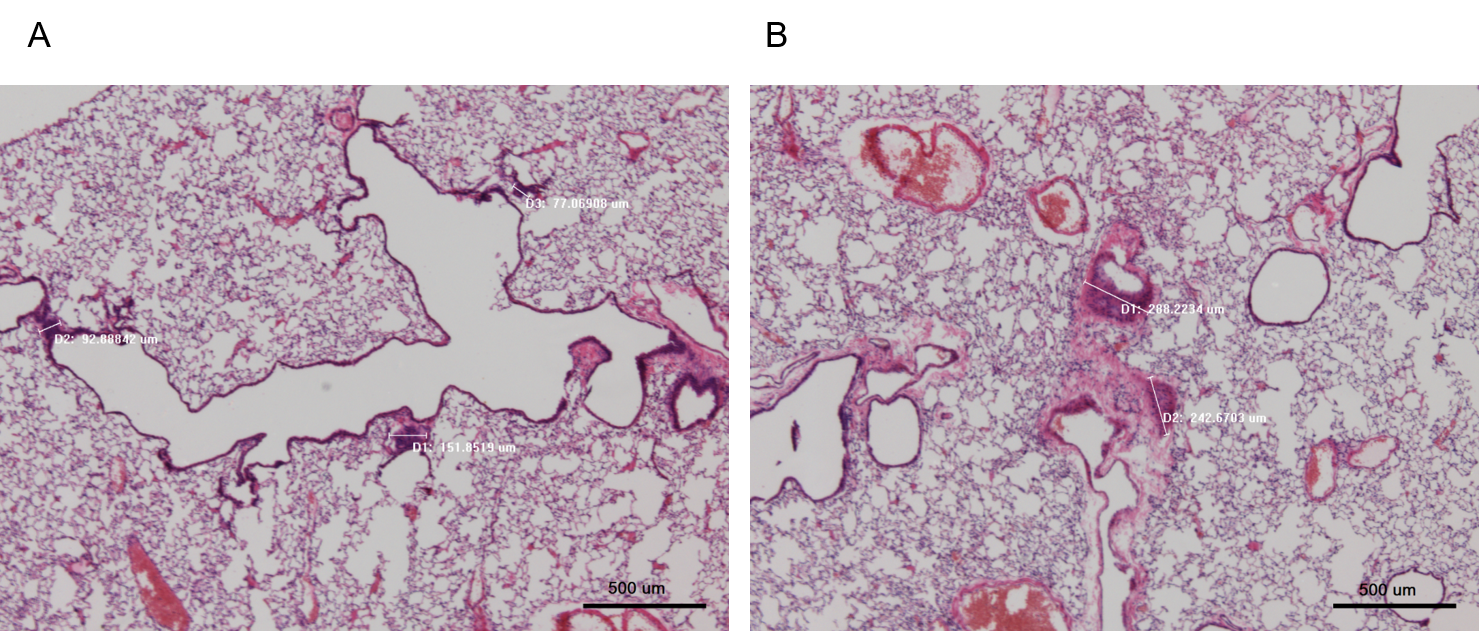


***Figure S11.*** Analysis of neoplasm size in lung tissues after tail vein injection of tumor cell lines. (A,B) Representative lung tissue sections (H&E) and identification of neoplastic lesions >120 μm in size.


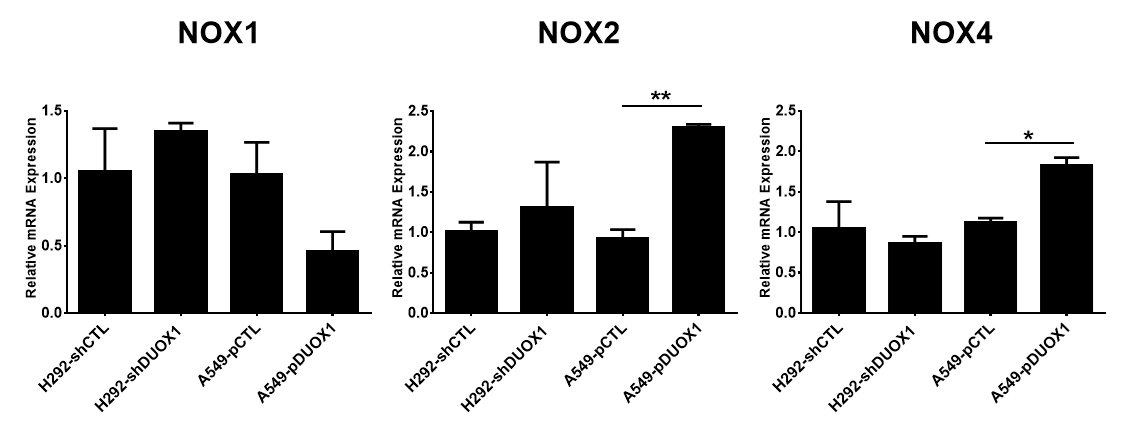


***Figure S12.*** Effect of DUOX1 silencing or overexpression on expression of NOX1,2 4. H292-shCTL, H292-shDUOX1, A549-pCTL and A549-pDUOX1 cell RNA extracts were analyzed for NOX1, NOX2, or NOX4 mRNA expression by qRT-PCR. Results are expressed as mean ± SD (n=4) *p<0.05, **p<0.01 were calculated by two-tailed Student’s T-test.

**Supplementary Table 1:** Evaluation of tumor cell engraftment into lung tissues by qPCR analysis of human Alu sequences.


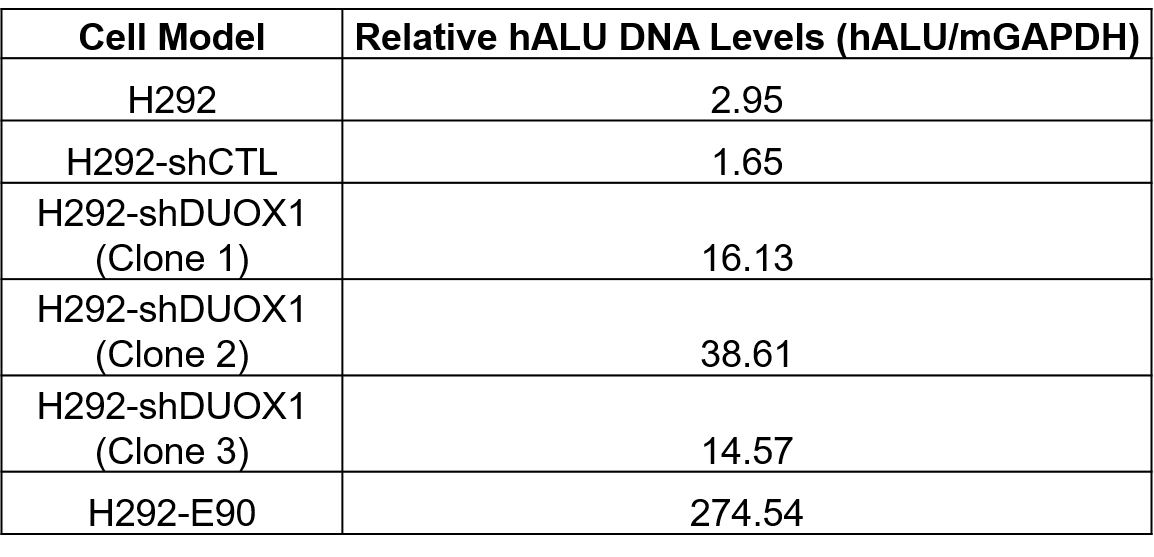


CD1 nude mice were injected with indicated tumor cell lines, and genomic DNA was extracted from whole mouse lung homogenates. Relative hALU levels were expressed compared to naïve mice.
